# Host defence related responses in bovine milk during an experimentally induced *Streptococcus uberis* infection

**DOI:** 10.1186/1477-5956-12-19

**Published:** 2014-04-11

**Authors:** Grant A Smolenski, Marita K Broadhurst, Kerst Stelwagen, Brendan J Haigh, Thomas T Wheeler

**Affiliations:** 1 Dairy Foods, AgResearch, Ruakura Research Centre, Private Bag 3123, Hamilton, New Zealand; 2 SciLactis Ltd, Waikato Innovation Park, Hamilton, New Zealand

**Keywords:** Bovine, Mastitis, Host defence, Mammary

## Abstract

**Background:**

Milk contains a range of proteins of moderate or low abundance that contribute to host defence. Characterisation of these proteins, the extent to which their abundance is regulated by pathogenic stimuli, and the variability of their response between and within individual animals would facilitate a better understanding of the molecular basis for this important function of milk.

**Results:**

We have characterised the host defence proteins in bovine milk and their responses to intra-mammary infection by a common Gram positive mastitis pathogen, *Streptococcus uberis*, using a combination of 2D gel electrophoresis and GeLC mass spectrometry. In total, 68 host defence-associated proteins were identified, 18 of which have a direct antimicrobial function, 23 of which have a pathogen-recognition function, and 27 of which have a role in modulating inflammatory or immune signalling. The responsiveness of seven proteins was quantified by western blotting; validating the proteomic analyses, quantifying the within- and between animal variability of the responses, and demonstrating the complexity and specificity of the responses to this pathogen.

**Conclusions:**

These data provide a foundation for understanding the role of milk in host-microbe interaction. Furthermore they provide candidate biomarkers for mastitis diagnosis, and will inform efforts to develop dairy products with improved health-promoting properties.

## Background

Milk is the sole source of nutrition for newborn mammals. In addition to providing a source of protein, carbohydrate and lipids to support growth, milk also contains a range of substances that contribute to the health and well-being of the developing offspring. For example, milk has long been known to provide passive immunity through the delivery of antibodies [1,2]. Also, milk contains proteins with antimicrobial properties, such as lactoferrin and lactoperoxidase [2], and immunomodulatory proteins and peptides [3]. In addition to protecting the neonate from infection, the milk immune factors also play an important role in the protection of the mammary gland itself [4]. Intra-mammary infections are prevalent in dairy cattle and often lead to clinical mastitis, which reduces milk yield and quality, is a significant cost to the dairy industry, and is an animal welfare issue. However, the molecular mechanisms through which the proteins in cows’ milk affect intestinal health in the neonate and protect against mastitis in dairy cows are not very well understood.

The application of proteomics approaches to the repertoire of milk proteins has resulted in the identification of a large number of proteins [5-9]. Among these, a significant number have functions associated with host defence or appear to be altered in abundance with infection of the mammary gland [6,9-12]. The responsiveness of a pre-selected group of 20 potential biomarkers to the Gram positive mastitis pathogen *Streptococcus uberis* has been studied [13]. However, the proteomic studies described to date have not addressed the range of milk proteins that are responsive to Gram positive pathogens, in particular *Streptococcus uberis*. The study described here aimed to address this by collecting milk from lactating cows that had selected quarters experimentally inoculated with *S. uberis*, preparing milk protein fractions, and applying two proteomics technologies in parallel; two-dimensional (2D) electrophoresis and gel electrophoresis-LC tandem mass spectrometry (GeLC). These analyses have identified 68 milk proteins with a host-defence related function, some of which have not been previously described in milk or as responsive to pathogenic stimulus. Some of these proteins have potential as biomarkers for diagnosis of mastitis in dairy cows.

## Results

### Two-dimensional electrophoresis of milk fractions

**Figure 1:**
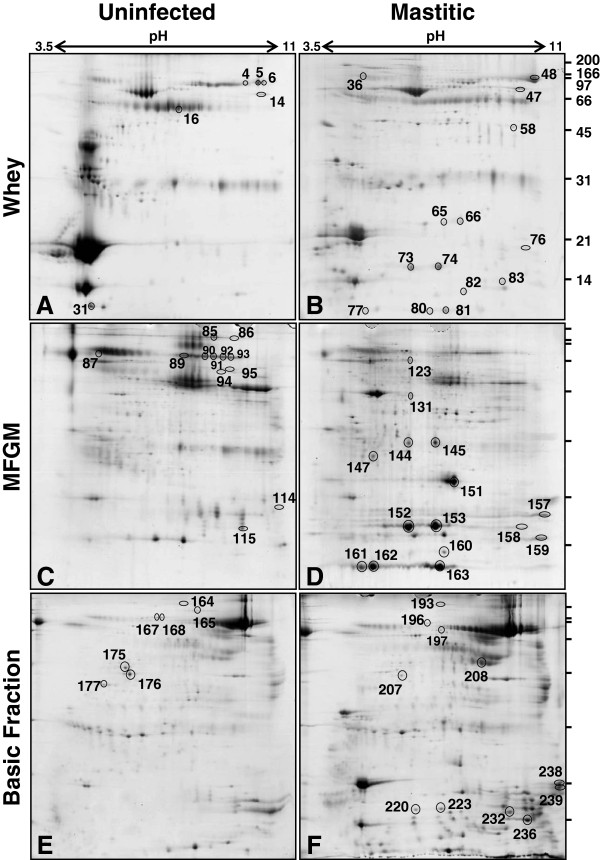
**Two-dimensional gel electrophoresis of bovine milk fractions.** Each gel was loaded with 350 μg of protein from whey **(A, B)**, milk fat globule membrane **(C, D)** or basic protein fraction **(E, F)** from either pooled milk from the infected quarters taken before inoculation **(A, C, E)** or from the same quarters taken at the time of diagnosis of clinical mastitis **(B, D, F)**. The gels were stained with Coomassie blue G250. The spots that were consistently altered greater than two-fold in abundance between the uninfected and mastitis gels, and that were identified as a host-defence related protein, are indicated. The number of each spot corresponds to that in the wider set of 240 spots subjected to MALDI-TOF analysis and listed in Additional file 2 and shown in Additional file 1.

Milk fractions produced from two pools, taken from clinically infected quarters after inoculation with *S. uberis* or from the same quarters before inoculation, were subjected to 2D electrophoresis, with each fraction being analysed in triplicate, resulting in 18 gels in total. Representative gels from each condition are shown in Figure 1. A total of 922 protein spots were assessed in the fractions (330 in whey, 424 in milkfat globule membrane (MFGM) and 168 in the basic protein fraction) using the PDQuest gel image analysis software package. This quantitative analysis resulted in 130 spots whose mean abundance between the groups was altered two-fold or greater. Among these, 56 spots were increased in abundance and 74 spots were decreased in abundance in response to infection. A total of 240 spots were chosen for identification by matrix-assisted laser desorption ionisation-time of flight mass spectrometry (MALDI-TOF). These included the 130 protein spots described above, as well as a further 110 spots that appeared to be their equivalent on other gels in the set. These 240 excised spots are shown in Additional file 1 and their identities and fold-changes listed in Additional file 2.

In some cases, multiple spots were found to represent alternate forms of the same protein. The 130 identified protein spots with altered mean abundance corresponded to 53 distinct gene products. These are listed in Table 1. The difference in abundance between the groups was assessed using the Student’s *T* test adjusted for multiple comparisons. The differences were found to be highly significant for 35 of the 53 gene products (p < 0.05), while another 6 were marginally significant (p < 0.10) as indicated on Table 1. Each of these proteins was classified into one of eight functional categories; major milk protein, chaperonins, host defence (see Additional file 3 for GO terms used), metabolism/homeostasis (GO:0008152, GO:0097009) structural, signalling (GO:0023052), transport (GO:0006810), and unknown, based on a combination of Gene Ontology classification and manual curation. A total of 23 out of the 53 proteins have a function associated with host defence. The 65 spots corresponding to these 23 proteins are indicated in Figure 1, labelled by the spot number as shown in Table 1.

**Table 1:** List of 53 proteins identified by 2DE-MALDI with mean abundance altered at least two-fold with mastitis

**Identified protein**	**Accession no.**	**Spot no.**	**Function**	**Fold change**	**p-value**^**†**^	**Fraction**
Actin, beta	AAI42414	104	Structural	3.04	**0.040**	MFGM
Actin, cytoplasmic 2	NP_001028790	130	Structural	20.93	**0.054**	MFGM
Adipose differentiation-related protein	ABM06155	106	Unknown	0.08	0.119	MFGM
Alpha-lactalbumin	CAA44927	110	Major milk protein	5.79	0.158	MFGM
Alpha-S1 casein	AAA30429	214	Major milk protein	0.16	**0.034**	BF
Alpha-S2 casein	CAH61065	219	Major milk protein	207.0	**0.002**	BF
Annexin A1	CAA39971	134	Structural	28.8	**0.020**	MFGM
Annexin A2	NP_777141	133	Structural	14.76	**0.027**	MFGM
Annexin A5	NP_001035567	138	Structural	5.16	0.116	MFGM
Apolipoprotein A-I	NP_776667	147	Host defence	8.12	0.144	MFGM
Beta casein	AAA30431	226	Major milk protein	18.0	**0.003**	BF
Beta-2-microglobulin	NP_776318	31	Host defence	0.06	**0.006**	Whey
Beta-lactoglobulin	732164A	109	Major milk protein	4.4	**0.005**	MFGM
Butyrophilin subfamily 1 member A1	P18892	96	Unknown	0.33	0.282	MFGM
Cathelicidin-1	DAA16881	220	Host defence	8.0	**0.001**	BF
Cathelicidin-7*	NP_777256	158	Host defence	37.54	**0.023**	MFGM
Chitinase-3-like protein-1	AAB64304	58	Host defence	47.8	**0.019**	Whey
Complement component C3	DAA27847	177	Host defence	0.004	**<0.001**	BF
Complement component C7 precursor	DAA17890	164	Host defence	0.41	*0.052*	BF
Endopin 1A	Q9TTE1	38	Metabolism	6.35	*0.072*	Whey
Enolase-1*	NP_776474	49	Metabolism	0.28	**0.005**	Whey
Fibrinogen	NP_001136389	55	Structural	0.09	**0.028**	Whey
Folate binding protein	1011184A	180	Metabolism	0.001	**<0.001**	BF
Gelsolin isoform b	NP_001029799	167	Host defence	0.24	**0.002**	BF
Glycosylation-dependent cell adhesion molecule-1	NP_777253	115	Host defence	0.45	**0.047**	MFGM
Haptoglobin	NP_001035560	65	Metabolism	74.1	**0.020**	Whey
Heat shock 70 kDa protein 5 (GRP 78)*	NP_001068616	117	Chaparonin	3.62	0.109	MFGM
Heat shock cognate 71 kDa protein	NP_776770	119	Chaparonin	7.58	*0.060*	MFGM
HRPE773	DAA15703	224	Unknown	8.09	**0.003**	BF
IgG_1_ heavy chain constant region	ABE68619	16	Host defence	3.6	0.353	Whey
IgM heavy chain constant region	AAB62251	87	Host defence	0.42	0.245	MFGM
Kappa casein	AF123251	183	Major milk protein	0.06	**0.001**	BF
Keratin, type II cytoskeletal 2 epidermal	XP_001254016	204	Structural	0.48	**0.029**	BF
Lactadherin (glycoprotein antigen MGP57/53)	S65138	97	Unknown	0.31	0.176	MFGM
Lactoferrin	AAA30610	48	Host defence	4.97	**0.040**	Whey
Lactoperoxidase	NP_776358	197	Host defence	3.07	**0.010**	BF
Pancreatic adenocarcinoma upregulated factor	DAA15702	236	Host defence	2.75	**0.003**	BF
Peptidoglycan recognition protein-1	NP_776998	239	Host defence	10.2	0.157	BF
Pigment epithelium-derived factor	NP_776565	174	Metabolism	0.51	**0.013**	BF
Polymeric immunoglobulin receptor	NP_776568	165	Host defence	0.05	**0.002**	BF
Quiescin Q6 sulfhydryl oxidase-1	NP_001095544	209	Metabolism	0.29	**0.030**	BF
S100 calcium-binding protein A12	P79105	81	Host defence	27.3	**0.029**	Whey
S100 calcium-binding protein A8	NP_001107197	160	Host defence	90.0	*0.092*	MFGM
S100 calcium-binding protein A9	NP_001039793	151	Host defence	9.14	*0.044*	MFGM
Serotransferrin	NP_803450	7	Transport	2.0	0.121	Whey
Serpin B1	Q1JPB0	52	Metabolism	0.47	**0.013**	Whey
Serpin B4*	NP_001095792	54	Metabolism	0.15	**0.029**	Whey
Serum albumin	NP_851335	172	Transport	6.11	**0.022**	BF
Serum amyloid A3	NP_851359	159	Host defence	10.03	**0.046**	MFGM
Serum amyloid A3.2 precursor*	XP_875660	76	Host defence	812.0	**<0.001**	Whey
Similar to serpin peptidase inhibitor, clade B like*	XP_001254097	57	Metabolism	0.31	0.261	Whey
Xanthine dehydrogenase	NP_776397	94	Host defence	0.12	**0.038**	MFGM
Zymogen granule membrane GP-2	NP_001069418	105	Host defence	0.4	*0.072*	MFGM

*denotes detection only by 2DE/MALDI-TOF.

^†^Results of the adjusted Students *T* test for significance of the difference from the three replicate gels per condition. Values in bold type are below 0.05, and those in italics are between 0.05 and 0.10.

### GeLC-MS of milk fractions

**Figure 2:**
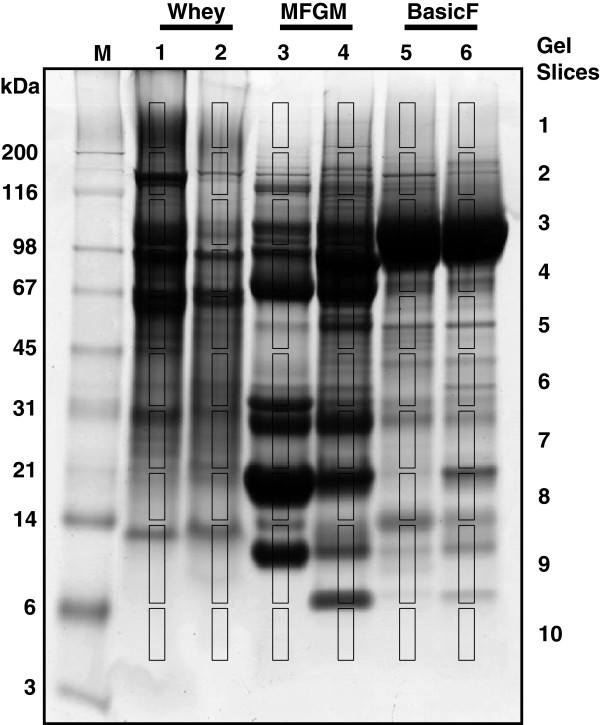
**SDS electrophoresis of milk fractions for GeLC analysis.** A total of 30 μg of each pooled milk fraction from the uninfected (lanes 1, 3, and 5) or mastitis (lanes 2, 4, and 6) milk samples was subjected to SDS electrophoresis and stained with Coomassie blue G250. A series of 10 slices were excised from each lane as indicated and each slice was subjected to in situ digestion, extraction of peptides followed by tandem mass spectrometry as described in the Methods section. The lesser intensity of lane 2 is most likely due to difference in protein/peptide composition between the infected and non-infected whey.

In order to obtain a more comprehensive identification of proteins, milk fractions were also analysed using an alternate method, GeLC. The Coomassie blue stained gel used for this analysis is shown in Figure 2. A total of 60 mass spectrometry (MS) runs (10 slices from each of 6 gel lanes) resulted in the identification of 189 distinct gene products. These are listed, along with their functional categorisation, in Additional file 3. All but six of the 53 gene products identified by 2D electrophoresis-MALDI-TOF (indicated with an asterisk in Table 1) were also identified by this GeLC approach. Combining both technical approaches, a total of 68 of the 195 proteins that were identified have a function associated with host defence. These are listed in Table 2. Among these, 18 are known to have antimicrobial activity, while 23 are involved in recognition of pathogens by the innate immune system and 27 are components of inflammatory or immune signalling. Sixteen of the proteins have not been reported as responsive to bacterial or lipopolysaccharde challenge in previous proteomic analyses of cows’ milk [6,9-11,14-18], and these are indicated in Table 2. Among these, four proteins (indicated in bold font in Table 2) have not previously been identified in milk.

**Table 2:** List of 68 host defence associated proteins identified in cows’ milk by either 2DE-MALDI-TOF or GeLC

**Spot #**^**a**^	**Protein name**	**Accession number**	**Condition**^**b**^	**Defence-related function**^**c**^
14	Alpha-2-HS-glycoprotein (Fetuin)	P12763	C/M	Mod. Infl. response
18^†^	Angiogenin-1	NP_001071612	C/M	Antimicrobial.
23	Apolipoprotein A-I	NP_776667	C/M	Mod. Infl. response
24	Apolipoprotein A-II	P81644	M	Mod. Infl. response
25	Apolipoprotein E	Q03247	C/M	Mod. Infl. response
**28**^**†**^	**Azurocidin-1**	DAA27509.1	C/M	Antimicrobial.
29	Beta-1,4-galactosyltransferase-1	P08037	C/M	Mod. Infl. response
30	Beta-2-microglobulin	NP_776318	C/M	Ant. Recog.
36	Cathelicidin* (2,6,7)	NP_777257	M	Antimicrobial.
37	Cathelicidin* (3,4,5,6,7)	NP_776935	M	Antimicrobial.
38	Cathelicidin-1	DAA16881	C/M	Antimicrobial.
39	Cathelicidin-2	AAA30404	M	Antimicrobial.
40	Cathelicidin-4	P33046	M	Antimicrobial.
41	Cathelicidin-7	NP_777256	M	Antimicrobial.
45	Chitinase-3-like protein-1	AAB64304	C/M	Mod. Infl. response
**50**^**†**^	**Common salivary protein/BSP30 b**	P79125	C/M	Antimicrobial
51	Complement 4 Binding Protein alpha	ABQ12991	C/M	Mod. Infl. response
52^†^	Complement 8, gamma polypeptide	NP_001103546	C	Antimicrobial.
53	Complement component C3	DAA27847	C/M	Ant. recog.
54	Complement component C4	NP_001159957	C/M	Mod. Infl. response
55	Complement component C5	NP_001160088	C	Antimicrobial.
56^†^	Complement component C7	DAA17890	C	Antimicrobial.
57	Complement factor B	NP_001035616	M	Mod. Infl. response
58^†^	Complement factor D	Q3T0A3	C/M	Mod. Infl. response
59	Complement factor H	Q28085	C	Mod. Infl. response
60^†^	Complement factor I	NP_001033185	C/M	Mod. Infl. response
83^†^	Follistatin	AAA30522	C	Mod. Infl. response
84^†^	Gamma glutamyl transferase 1	DAA20447	C/M	Mod. Infl. response
85	Gelsolin	NP_001029799	C/M	Mod. Infl. response
87	GlyCAM-1	NP_777253	C/M	Ant. recog.
100	IgA heavy chain constant region	AAC98391	C/M	Ant. recog.
101	IgG_1_ heavy chain constant region	ABE68619	C/M	Ant. recog.
102	IgM heavy chain constant region	AAB62251	C/M	Ant. recog.
103	Immunoglobulin J chain	AAI03427	C/M	Ant. recog.
104	Ig kappa chain variable region	XP_002691399	C/M	Ant. recog.
105	Ig kappa light chain variable region	DAA24659	C/M	Ant. recog.
106	Ig lambda chain variable region	AAI51501	C	Ant. recog.
107	Ig lambda light chain constant region	AAI42356	M	Ant. recog.
108	Iglambda light chain variable region	AAI02190	C/M	Ant. recog.
109	Ig lambda-like polypeptide-1	NP_001077269	C/M	Ant. recog.
110	Inter-alpha (globulin) inhibitor H4	Q3T052	M	Mod. Infl. response
125	Lactoferrin	AAA30610	C/M	Antimicrobial.
126	Lactoperoxidase	NP_776358	C/M	Antimicrobial.
129	Lipocalin 2	XP_605012	C/M	Antimicrobial.
130	Lipopolysaccharide-binding protein	Q2TBI0	C/M	Ant. recog.
133^†^	Mannose-binding protein C	O02659	C	Ant. recog.
134	Mucin-1	Q8WML4	C/M	Antimicrobial.
136	Myeloperoxidase	NP_001106769	M	Antimicrobial.
139	Osteopontin	P31096	C/M	Mod. Infl. response
**140**^**†**^	**PAUF**	XP_002697974	C?M	Mod. Infl. response
141	Peptidoglycan recognition protein-1	NP_776998	C/M	Ant. recog.
142	Peptidyl-prolyl cis-trans isomerase A	P62935	C/M	Mod. Infl. response
143^†^	Peptidyl prolyl cis-trans isomerase B	P80311	C/M	Mod. Infl. response
147	Platelet glycoprotein-4 (PAS IV/CD36)	P26201	C/M	Ant. recog.
148	Polymeric immunoglobulin receptor	NP_776568	C/M	Ant. recog.
150	Prostaglandin D2 synthase 21 kDa	O02853	C/M	Mod. Infl. response
164	S100 calcium-binding protein A12	P79105	C/M	Mod. Infl. response
165	S100 calcium-binding protein A8	NP_001107197	M	Mod. Infl. response
166	S100 calcium-binding protein A9	NP_001039793	C/M	Mod. Infl. response
173	Serum amyloid A3	ACL13307	M	AP response/Ant. recog.
174^†^	Serum amyloid A3.2	XP_875660	C/M	AP response/Ant. recog.
**175**^**†**^	**Serum amyloid P-component**	Q3T004	C	AP response/Ant. recog.
185^†^	Thrombospondin-1	Q28178	C/M	Mod. Infl. response
187^†^	Transforming growth factor beta-2	P21214	C/M	Mod. Infl. response
192	Vitamin D-binding protein	Q3MHN5	C/M	Mod. Infl. response
193	Xanthine dehydrogenase/oxidase	NP_776397	C/M	Antimicrobial.
194	Zinc-alpha-2-glycoprotein	Q3ZCH5	C/M	Ant. recog.
195	Zymogen granule membrane GP-2	NP_001069418	C/M	Ant. recog.

*denotes a peptide that maps to multiple members of the protein family, as listed in parentheses.

^a^Spot numbers match those from Additional file 2.

^b^C = control; M = mastitic.

^c^Ant. Recog. = antigen recognition; AP response = acute phase response; Mod. Infl. response = modulates inflammatory response.

Entries in bold text denote proteins not previously reported as present in milk.

^†^denotes not previously reported as changed in abundance in response to infection.

### Quantification of responsiveness of selected milk proteins

**Figure 3:**
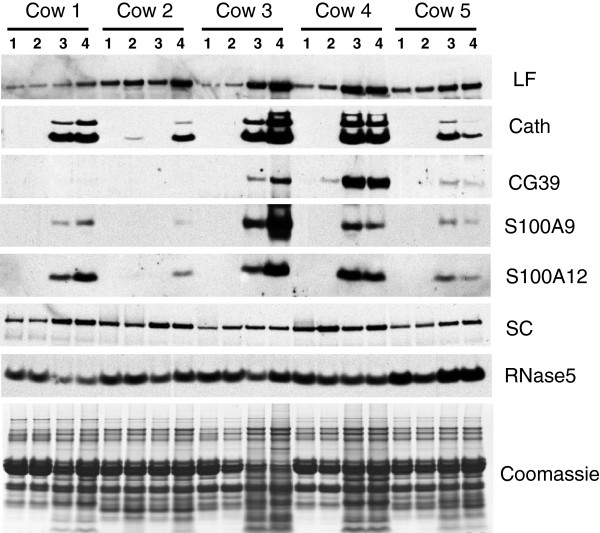
**Western blotting of individual milk samples.** Portions of each milk sample were diluted 15-fold in sample buffer and subjected to SDS electrophoresis followed by western blotting using antibodies raised against lactoferrin (LF), the cathelin domain of cathelicidin-1 (Cath), CG39, S100A9, S100A12, secretory component (SC), and RNase5. Peroxidise-conjugated goat anti-rabbit IgG was used as secondary antibody. The signals were visualised by enhanced chemiluminescence followed by exposure to X-ray film. Quarters for each cow were untreated (1), mock inoculated (2), or infused with *S. uberis* (3 and 4). A replicate gel was stained with Coomassie blue (bottom panel).

For logistical reasons it was necessary to perform the analyses described above on pooled milk, as analyses on individual milk samples would require 162 2D gels and 540 LC-tandem MS runs. Therefore, in order to assess variation among the individual milk samples in the magnitude of the response to infection, as well as the difference between udder quarters within the same animal or between different animals, western blotting was performed for seven of the host defence associated proteins identified above. These were lactoferrin (CAA38572), cathelicidin (the antibody detects all bovine cathelicidins), chitinase-3-like protein-1/CG39 (AAB64304), S100A9 (NP_001039793), S100A12 (P79105), secretory component/polymeric immunoglobulin receptor (P81265), and RNase5/angiogenin (NP_001071612). These proteins were selected to provide examples of different types of host-defence related proteins for which there is prior knowledge of their responsiveness to pathogens or their function. Skim milk, taken from each quarter at the time of diagnosis of clinical mastitis in the cow, was analysed (Figure 3). Quantification of the signals produced fold changes that were consistent with that obtained for the 2D analysis as shown in Table 1. These changes, along with their statistical significance are shown in Table 3. Lactoferrin (5.0- and 6.8-fold by 2DE and western analyses, respectively), cathelicidins (37- and 30-fold), chitinase-3-like protein-1/CG39 (48- and 15.5-fold), S100A9 (12.5- and 29-fold) and S100A12 (3.6- and 39-fold) all were increased in abundance. Secretory component was observed to be decreased in the 2D gels (0.05-fold change) but was not significantly altered in the western analysis. Although a moderate but significant increase was observed in the mastitic samples from two of the cows, the change was not significant when data all the cows was considered. RNase5/angiogenin was not altered greater than 2-fold in abundance in the 2D gels and therefore was not included in Table 1, however a moderate but significant decrease (to 0.6 of the pre- infection abundance, corresponding to a 1.7-fold change) was observed for this protein in the western blot analysis.

**Table 3:** Mean abundance of selected host defence proteins

**Protein**	**Uninfected**	**Mastitis**	**Fold change**	P value*
**Lactoferrin**	8.2	55.2	6.8	7 × 10^-4^
**Cathelicidin**	3.3	100.0	30.1	2 × 10^-8^
**CG39**	0.6	8.6	15.5	3 × 10^-3^
**S100A9**	3.3	95.0	29.2	8 × 10^-4^
**S100A12**	3.0	118.0	39.0	7 × 10^-7^
**Secretory component**	65.0	53.3	0.8	0.124
**RNase5**	77.6	47.6	0.6	4 × 10^-5^

Data is the mean of nine individual quarters.

*Probability that the difference between the conditions has arisen by chance, as determined by ANOVA.

**Figure 4:**
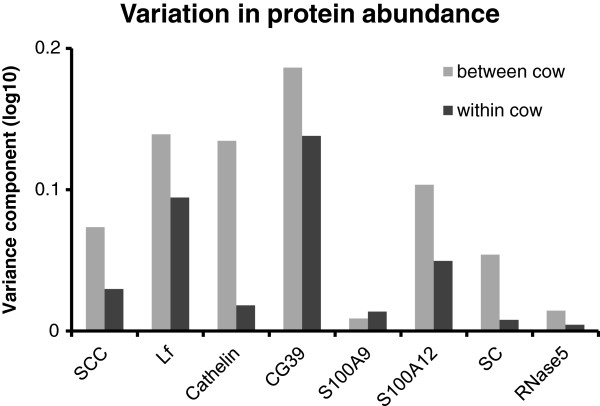
**Variance analysis of the abundance of specific host-defence proteins in milk.** Chemiluminescence signals, captured directly from western blots using a CCD camera, as well as the somatic cell counts (SCC), were subjected to ANOVA. The variance components for within-cow and between-cow groupings are shown for the SCC and each of the antibody signals.

**Figure 5:**
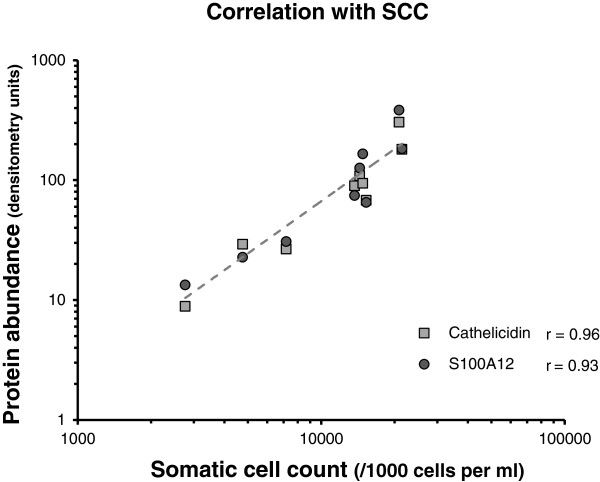
**Correlation analysis of cathelicidin and S100A12 with somatic cell count.** The abundance of cathelicidin and S100A12, as determined by densitometry of western blots of the mastitic milk samples, was plotted against the somatic cell count. The correlation coefficient (r) of each protein with SCC was determined from the plot using the statistical function in Excel. The trendline for both proteins overlie one another and are depicted as a dashed line.

The abundance of some of the proteins varied to a large extent among the individual milk samples, despite the quarters exhibiting very similar clinical signs in response to an identical microbial challenge at the time of collection. The variability in the abundance of the seven proteins, as well as somatic cell count (SCC), between the infected quarters was therefore determined. The variability was significantly higher between infected quarters from the different cows (P < 0.01), compared to infected quarters from the same cow (Figure 4). Three of the proteins measured; cathelicidin, S100A9 and S100A12, were particularly highly responsive to mammary infection. The level of these proteins in milk was compared to the SCC in the same quarter. Abundance of both cathelicidin and S100A12 were highly correlated with SCC (Figure 5), with correlation coefficients of 0.96 and 0.93, respectively. The abundance of S100A9 was less well correlated but nevertheless significant (r = 0.69; data not shown).

## Discussion

This study has identified the products of 195 distinct genes in cows’ milk; 6 major milk proteins plus 189 minor milk proteins, a similar number to that reported in several previous proteomic studies of milk [5-9,11,12,14,19]. An even greater complexity of milk was described one recent investigation that reported nearly 3000 distinct proteins, although most of these (2350) were present in one extremely highly purified fraction, exosomes [9]. The degree of concordance between the proteins listed in the current study and the earlier reports suggests that the large majority of the biologically significant host defence proteins in cows’ milk have been identified.

The identification of as many as 68 host-defence associated proteins in this study suggests that milk has a significant host-defence function and that *S uberis* is capable of eliciting a host defence response as complex as that for other mastitis pathogens; significantly greater than the 20 proteins previously analysed for responsiveness to this pathogen [13]. Many (16 of the 53 proteins listed in Table 1 and 27 of the 68 proteins listed in Table 2) were also identified as responsive to another Gram positive pathogen, *S aureus*[9] despite differences in the experimental design and analysis techniques. Additionally, a distinct set of 16 out of the 53 proteins listed in Table 1 were also identified in an earlier study of *E coli* induced responses which used a similar experimental design and analysis technique [14]. These comparisons suggest that common pathways may be activated in response to each pathogen. In the current study, sixteen of the host defence proteins listed in Table 2 have not previously been identified as responsive to inflammatory stimuli in milk, and four, to our knowledge, have not been reported in previous proteomic studies of milk. These are azurocidin 1 (DAA27509.1), pancreatic adenocarcinoma upregulated factor (PAUF) (XP_002697974), common salivary protein BSP30b (P79125), and serum amyloid P-component (Q3T004). Azurocidin is a neutrophil granule protein with potent antimicrobial activity [20]. PAUF is an endothelial cell activator and lectin that interacts with Toll-like receptor 2 (TLR2) and stimulates inflammatory signalling [21]. BSP30b is a member of a family of proteins related to bactericidal/permeability increasing protein that is found in bovine saliva [22] and has modest antimicrobial activity [23]. Serum amyloid P component is an acute phase response protein that is a member of the pentraxin family of pathogen-recognition proteins [24]. The host defence protein repertoire of milk revealed here has similarities to that of other mucosal secretions that have a host defence function, such as saliva, tears and reproductive tract secretions [25]. For example, reproductive tract secretions also contain lactoferrin as well as members of the cathelicidin and S100 families of proteins [26].

Several functional sub-categories of host-defence proteins were identified. Approximately a third (18 out of 68) appear to function as effector proteins through their antimicrobial activity. These include; lactoferrin, which is secreted from mammary epithelial cells as well as being present in neutrophils; a range of neutrophil granule proteins such as azurocidin and cathelicidins; several members of the complement family; and the antimicrobial oxidases, lactoperoxidase, myeloperoxidase and xanthine oxidase. Approximately another third (23 of the 68) appear to function as pathogen-recognition proteins, and presumably help initiate a pathogen-specific immune response. These include some well characterised pathogen-recognition proteins such as the immunoglobulins, complement C3, and lipopolysaccharide-binding protein. Other less well-characterised such proteins include peptidoglycan recognition protein (NP_776998), mannose-binding protein C (O02659), chitinase-3-like protein-1/CG39 (AAB64304) and common salivary protein BSP30b (P79125). Some of these proteins function as lectins [21,27], suggesting that recognition of carbohydrate moieties is an important aspect of the host defence function of milk. One of these proteins, (serum amyloid P-component) is identified for the first time in milk. Approximately a final third (27 out of 68) appear to function as modulators of the inflammatory response. These include transforming growth factor β [28], S100 family members [29], complement family members [30], and members of the apolipoprotein family [31]. Collectively, all of these types of proteins appear to confer on milk the property of facilitating responses to a wide range of exogenous substances. In the gut, one possibility is that the proteins could play a role in either the defence against enteric pathogens or, conceivably, in the development of oral tolerance to food-derived antigens. Thus these proteins could complement the function of the proposed probiotic and prebiotic components of milk in optimal digestive function and development of the immune system in the neonate.

The identification of intracellular proteins in this as well as previous studies [5-8,11,14,19] might be considered surprising. These proteins may originate from mammary epithelial cells that have been sloughed off the tissue, from macrophages resident in the apical space, from neutrophils which accumulate in large numbers in milk during mastitis, or from cytoplasmic crescents that are incorporated into the milk fat globule during its formation at the apical surface of the mammary epithelial cells. In total, 38 of the 195 proteins identified in this study are known to be present in neutrophils [32], suggesting these cells are a significant contributor to the proteome of mastitis milk.

The use of a pooling approach allowed information to be gained from a large set of individual samples while minimising the number of analyses, but has the potential also to obliterate responses that are particular to individual animals. The proteomic analyses were therefore accompanied by analysis of selected proteins in individual samples using a “gold standard” quantitative technique, immunoblotting. This approach allowed direct comparison of the changes obtained by proteomic analysis of pooled samples with those obtained by immunoblotting, confirming the validity of the approach for six out of the seven proteins. The quantitative analysis revealed a wide variety in the extent of responses to the presence of a pathogen. The cathelicidins and S100 proteins were the most responsive proteins observed. Both proteins are abundant antimicrobial neutrophil proteins (with some expression in epithelial) and their increased level in mastitic milk is likely the result of neutrophil invasion and degranulation [33-35]. These proteins could have utility as biomarkers for the early diagnosis of infection. We have previously shown that cathelicidins correlate highly with SCC in the early stages of inflammation [36]. The abundance of CG39 was increased with infection but to a lesser extent. This chitin-binding protein, which is produced by mammary epithelial cells and secreted into milk during an inflammatory response [37], could conceivably facilitate recognition of chitin-containing fungal pathogens. In contrast, the abundance of RNase5 decreased moderately with infection. This antimicrobial protein [38], which is secreted from mammary epithelial cells as well as a range of additional tissues, may also facilitate inflammatory signalling in response to nucleic acids [39]. Down regulation of this protein, if indeed it is biologically significant, could conceivably provide a feedback loop to regulate inflammation. Secretory component is a subunit of secretory IgA which is also found in free form in some mucosal secretions and has been reported to influence microbial colonisation and inflammatory signalling [40]. Several 2D spots correspond to secretory component, and infection results in a decrease in only a subset of them. The unchanged abundance in the western blot analysis suggests that infection results in post-translational modification of the protein without altering its overall abundance. Lactoferrin is secreted into milk by mammary epithelial cells at elevated levels during an inflammatory response and is thought to act as an effector protein through sequestering ferrous ions [41] and the generation of an antimicrobial peptide, lactoferricin [42]. The increase observed here is consistent with other proteomic studies [15].

Taken together, the data underscore the highly individual nature of the inflammatory response, even between quarters of the same cow, despite each being given an identical bacterial inoculation and taken to the same clinical endpoint. The variability in response of these proteins and perhaps others identified in this study could conceivably be exploited for improved diagnostics, for example to determine the nature of the pathogen or stage of infection, or the degree of susceptibility of the cow to mastitis. The data also show that a Gram positive mastitis pathogen is capable of eliciting an inflammatory response that is as complex as that previously reported for the Gram negative pathogen, *E coli*[14]. Understanding of mucosal host defence is incomplete, and determination of the differences in responses to a range of pathogens and commensal species may shed light in understanding the complex interaction between the host organism and pathogens at the molecular level. Besides lactoferrin, which is already being extracted commercially [43], other host defence related proteins could conceivably be a focus for commercial extraction. Also, the composition of milk could be optimised for host-defence functionality through the on-farm modulation of the composition of milk. Thus, this work will provide a resource for understanding the physiological consequences of, and optimising the health benefits from consumption of bovine milk by humans.

## Methods

### Collection of uninfected and mastitic milk samples

Milk samples were obtained from 10 healthy non-pregnant Friesian-Jersey cross-bred cows in late lactation (239 ± 24 days in milk). These cows were grazed on pasture and ranged in age from 3 to 8 years. The animals were inoculated in the two rear quarters with 800 colony-forming units (cfu) of *Streptococcus uberis* strain O140J using a procedure as previously described [36]. Approval was obtained from the local Animal Ethics committee. Mean milk SCC and standard error for all bacterial-challenged quarters at the start of the study was 33,800 ± 7,090 cells/mL. SCC was determined using a commercial automated fluorescent microscope somatic cell counter (Fossomatic, Denmark) operated by Livestock Improvement Corporation, Hamilton, New Zealand. The front right quarter was inoculated with vehicle only and the front left quarter was left untreated. Milk samples were collected from all four quarters at 0, 24, 30, 48, 54, 72, 78 and 96 h post-infusion or until clinical signs of mastitis were apparent; either red or swollen quarters, discoloration, or clots in the milk, which were scored on a 1–5 scale using the California Mastitis Test (CMT) system [44]. Nine of the 20 inoculated quarters produced a CMT score of 3 or greater and had one or more signs of clinical mastitis (tenderness, elevated temperature or swelling of the udder). For these the mean SCC was 12.8 × 10^6^ cells/mL with a range of 2.7 × 10^6^ to 21.4 ×10^6^ cells/mL. Milk was collected aseptically from each of the nine clinically infected quarters at the time when that quarter was first diagnosed and subjected to microbiological analysis. The quarters were then milked out using quartermilkers (Shoof International Ltd, Cambridge, New Zealand) and the milk used for SCC determination, protein fractionation and proteomic analysis. All animals were treated with antibiotics at the end of the trial.

### Preparation of milk fat globule membrane, whey and basic protein fractions

Whole milk samples were mixed with phenylmethanesulphonyl fluoride to a final concentration of 2 mM to minimise proteolysis. For the preparation of MFGM and whey, two pooled milk samples were created by mixing together the milk from the nine infected quarters collected at the time of positive CMT, and milk from the same quarters collected before the inoculations. These were centrifuged at 1,500 × g at 4°C for 20 min, and the fat layer removed. The fat layer was washed six times with phosphate buffered saline to remove unbound casein and somatic cells. The washed fat was then incubated for 1 h at room temperature in buffer containing 50 mM Tris–HCl, pH 6.8, 150 mM dithiothreitol, 20% glycerol, and 4% sodium dodecyl sulphate (SDS) at a concentration of 1 mL/g of fat with periodic vortexing. This was followed by incubation in a boiling water bath for 4 min. The fat layer was then removed from the aqueous layer by two successive centrifugations at 12,000 × g for 10 min.

The MFGM proteins were recovered from the solution using a modification of a previously described chloroform/methanol precipitation method [45]. The sample (100 μL) was mixed sequentially with 400 μL of methanol, 100 μL of chloroform and 300 μL of water and then centrifuged at 9,000 × g for 1 min. The resulting protein pellet was then resuspended in 300 μL of methanol and centrifuged at 9,000 × g for 2 min. The pellet was air dried, then resuspended in a solution containing 8 M urea, 3 M thiourea, 20 mM Tris, pH 7.5, 2% 3-[(3-cholamidopropyl)dimethylammonio]-1-propanesulfonate (CHAPS) and 0.4% dithiothreitol for subsequent 2D electrophoresis. Protein concentration was assessed using the RC-DC Protein Assay (Bio-Rad, Hercules, CA, USA).

To produce whey, the skimmed milk was centrifuged at 100,000 × *g* for 1 h at 4°C to pellet the casein micelles, the supernatant was removed and centrifuged a second time, and the translucent supernatant from the second centrifugation was collected and stored at -20°C.

The basic protein fraction was generated by passing skimmed milk, pooled as described above, over a SP-Sepharose Fast Flow (GE Healthcare, Piscataway, NJ, USA) cation exchange column. Bound proteins were eluted with 1 M NaCl and then subjected to tangential flow filtration with a 10 kDa cut-off membrane (Select, GE Healthcare) to 4 μS/cm. The final eluate was freeze-dried and the powder stored at room temperature for future analysis.

### Two-dimensional electrophoresis

Uninfected and mastitic milk fractions were analysed in triplicate by 2DE using previously described procedures [6]. Briefly, 350 μg of protein was loaded onto pH 3.5-11 immobilised pH gradient strips (GE Healthcare) and focused for 95 kVh. After equilibration, the strips were loaded onto 14% acrylamide SDS polyacrylamide gel electrophoresis (SDS-PAGE) gels for second dimension separation. The resulting gels were stained with colloidal Coomassie blue, scanned using a densitometer (GS800, Bio-Rad) and the images captured using the QuantityOne software package (Bio-Rad). Protein spots were quantified using the PDQuest analysis software package (version 8.0.1, Bio-Rad). Each gel was normalised for variations in loading using the locally weighted scatterplot smoothing (LOESS) regression model algorithm in PDQuest. The fold change was determined by dividing the average intensity of each spot from the mastitic gels with the average intensity of its matched spots from the uninfected gels. Spots consistently showing at least a two-fold difference in the mean abundance between the conditions were excised and subjected to MALDI-TOF mass spectrometry. An adjusted p value for the difference between uninfected and infected pools for each protein was determined using the Student’s *T* test function in Excel on the log transformed data. An adjustment was then made for multiple comparisons, using the p.adjust function in R (http://r-project.org, version 3.0.2) employing the FDR method [46].

### MALDI-TOF mass spectrometry

Excised spots were washed three times with 25 mM ammonium bicarbonate in 50% (v/v) acetonitrile and subsequently vacuum-dried. The spots were then rehydrated with the addition of 10 μL of a solution containing 25 mM ammonium bicarbonate, 0.1% (w/v) *n*-octyl-β-D-glucopyranoside and 40 ng of sequencing grade tosyl phenylalanyl chloromethyl ketone (TPCK)-modified porcine trypsin (Promega, Madison, WI, USA). After 30 min of incubation at 4°C a further 20 μL of 25 mM ammonium bicarbonate was added to cover the gel pieces, and the mixture incubated overnight at 37°C. The peptides were then extracted, lyophilized and reconstituted in a solution containing 2% (v/v) acetonitrile and 0.1% (v/v) trifluoroacetic acid (TFA).

A 1.2 μL portion of each peptide extract was mixed with 0.6 μL of 10 mg/mL α-cyano-4-hydroxycinnamic acid, prepared in 67% acetonitrile/0.5% TFA, and spotted onto an AnchorChip MALDI plate (Bruker Daltonics, Bremen, Germany) using the dried droplet technique. After drying, the samples were recrystallised by the addition of 0.5 μL 80% (v/v) ethanol/0.1% (v/v) TFA. Mass spectrometry was performed on an autoflex II MALDI-TOF/TOF (Bruker) in reflector mode. External mass calibration was performed using peptide calibration standards (Bruker). The Biotools 3.0 program (Bruker) was used to convert spectra in the *m/z* range 500 to 3500 into peak lists which were then used to perform a peptide mass fingerprint search against the National Center for Biotechnology Information (NCBI) (17-6-2010) non-redundant database, restricted to *Bos taurus* using the Mascot search engine (version 2.2, Matrix Science, London, UK). The following search parameters were used; trypsin was selected as the protease with a maximum of one missed cleavage site, taxonomy was set to mammalia, peptide mass tolerance was set to ±100 ppm, carbamidomethylation of cysteine residues (+57.03 Da) was set as a fixed modification and oxidation of methionine residues (+15.99 Da), and phosphorylation of serine and threonine residues (+79.9663 Da) were set as a variable modification. Proteins with Mascot scores of 67 and above (P < 0.05) were considered to be positive identifications. The identified proteins are listed in Additional file 2, along with their Mascot scores and number of peptides identified. The identified proteins were assigned to one of eight functional categories (major milk protein, chaperonins, host defence, metabolism/homeostasis, structural, signalling, transport, and unknown) based on a combination of Gene Ontology classification and manual curation.

### GeLC analysis

Each sample was dissolved in SDS electrophoresis sample buffer, heated in a boiling water bath for 3 min, and 30 μg of total protein was resolved by SDS-polyacrylamide gel electrophoresis using a 12% Bis Tris gel (Criterion XT, Bio-Rad). The gel was fixed in 50% (v/v) methanol/2% (v/v) phosphoric acid for 30 min, reduced using 10 mM dithiothreitol at 65°C for 1 h and alkylated with 45 mM iodoacetamide for 1 h at ambient temperature in the dark. The protein bands were visualised with colloidal Coomassie G-250. The central portion of each lane was cut into 10 contiguous slices and each slice was destained and incubated overnight in 1.5 μg of modified porcine trypsin (Promega) in 80 μL of 50 mM ammonium bicarbonate, pH 7.8. The peptides were extracted, lyophilized and reconstituted with 30 μL of a solution containing 0.5% (v/v) formic acid and 5% (v/v) acetonitrile.

A portion of each sample (8 μL) was subjected to reversed-phase liquid chromatography coupled online with an LTQ linear ion trap mass spectrometer utilizing a nanospray electrospray ionisation interface (Thermo Fisher Scientific Inc., San Jose, CA, USA) as previously described [47]. Data-dependent tandem MS analysis of peptide ions was performed using the “triple-play” method with dynamic exclusion enabled. Tandem mass spectra were extracted and analysed using the SEQUEST program within the BioWorks software package (v3.3.1 SP1, Thermo Scientific, San Jose, CA). The data files were searched against the NCBI non-redundant database (20-6-2010) restricted to the following seven species; *Bos taurus*, *Ovis aries*, *Capra hircus*, *Mus musculus*, *Rattus rattus*, *Homo sapiens*, and *Sus scrofa* (497206 entries, ftp://ftp.ncbi.nih.gov/blast/db/FASTA). Carbamidomethylation of cysteine residues (+57.0215 Da) was set as a fixed modification, while oxidation of methionine (+15.9949 Da), and phosphorylation of serine, threonine and tyrosine residues (+79.9663 Da) were set as variable modifications. Trypsin was selected as the cleavage protease allowing up to two missed cleavages sites. Protein identifications were filtered using the following peptide criteria: (i) P(pep) value lower than 0.0005; (ii) cross-correlation values higher than 1.8, 2.5 or 3.5 for singly, doubly or triply charged peptides, respectively; (iii) the percent ion coverage greater than 30%; and (iv) two or more unique peptides detected from the protein. For the evaluation of false discovery rate FDR) a composite database containing the original forward sequence as well as its corresponding reversed sequence was created and searched. The FDR was calculated using the equation: FDR (%) = (2*n(rev))*100/(n(rev) + n(fwd)), where n(fwd) and n(rev) are the number of peptides identified using the above filtering criteria, from the forward and reversed sequences respectively. The reversed sequences were identified by tagging their FASTA protein descriptors. The FDR value obtained was 2.94%. For proteins identified through only a single peptide hit (singleton), an ion coverage stringency of 65% for doubly charged peptides and 43% for triply charged peptides was applied. All singletons meeting these criteria were included. These additional requirements resulted in the elimination of all reversed sequence hits and some forward sequence matches with poor spectra. The Gene Ontology identifiers and descriptors were obtained for each of the identified proteins, and a host-defence function was assigned based on linkage with one of the following four functional terms; complement activation (GO:0006953), defence response (GO:0006956), immune response (GO:0006955), or inflammatory response (GO:0006954). The functional assignments of all the proteins were then manually curated, resulting in deletion of two proteins (acid 1 glycoprotein and haptoglobin) due to lack of published evidence for a host-defence function, and addition of 48 others as having a host defence function. The Gene Ontology and literature evidence for these assignments are included in Additional file 3.

### Western blot analysis

A 25 μg portion of skim milk from individual quarters, prepared in SDS electrophoresis sample buffer, was subjected to electrophoresis using a 12% Bis-Tris gel (Criterion XT, Bio-Rad) then transferred to nitrocellulose. The blots were blocked with 4% (w/v) non-fat milk powder and probed with rabbit polyclonal antibodies against either bovine lactoferrin, the cathelin fragment of the bovine cathelicidins, CG39, S100A9, S100A12, secretory component, or RNase5, using previously described procedures [36]. Previous analyses demonstrate that the abundance of these proteins in the milk-based blocking solution is too low to cause interference with signal detection [36]. The anti-lactoferrin antibody was raised against full length protein and was a gift from John Tweedie (Massey University, Palmerston North, New Zealand), while the remaining antibodies were raised in-house in rabbits, using either full length recombinant protein (CG39), native purified protein (secretory component), or 10-15-mer synthesised peptides coupled to Keyhole limpet hemocyanin (S100A9, S100A12 and RNase5) as immunogens. The immunoreactive protein bands were visualized by enhanced chemiluminescence with a 1/15,000 dilution of horseradish-peroxidase-conjugated anti-rabbit immunoglobulin G raised in goats (Sigma, St. Louis, MO) and digitally quantified using a charged couple device camera (Chemidoc XRS, Bio-Rad). The data was captured using QuantityOne software. The integrated signal density was converted to an amount of protein relative to a standard, and the fold change was then determined.

## Abbreviations

2D: Two-dimensional; Cfu: Colony forming units; CHAPS: 3-[(3-cholamidopropyl)dimethylammonio]-1-propanesulfonate; CMT: California mastitis test; FDR: False discovery rate; GeLC: Gel electrophoresis-LC tandem mass spectrometry; LC: liquid chromatography; LOESS: Locally weighted scatterplot smoothing; MALDI-TOF: Matrix assisted laser desorption ionisation time of flight mass spectrometry; MFGM: Milk fat globule membrane; MS: Mass spectrometry; NCBI: National Center for Biotechnology Information; PAUF: Pancreatic adenocarcinoma upregulated factor; SCC: Somatic cell count; SDS: Sdium dodecyl sulphate; SDS-PAGE: Sodium dodecyl sulphate polyacrylamide gel electrophoresis; TFA: Trifluoroacetic acid; TLR2: Toll-like receptor 2; TPCK: Tosyl phenylalanyl chloromethyl ketone; GO: Gene ontology.

## Competing interests

The authors declare they have no competing interests.

## Authors’ contributions

TTW, KS and GAS conceived and designed the study with input from BJH and MKB. Animal manipulations and sampling, 2DE, and MS analysis was carried out by GAS. Western blotting was carried out by GAS and MKB. Data analysis and interpretation was carried out by GAS, TTW and BJH. The manuscript was written by TTW, with significant input from all authors. All authors read and approve the final manuscript.

## Supplementary Material

Additional file 1 **Representative 2D gel images of uninfected and mastitic whey, MFGM and basic proteins.** The spots indicated with numbers depict those spots subjected to MALDI-TOF analysis. The spot numbers correspond to those listed in Additional file 2. Click here for file

Additional file 2 **Spots excised from the 2D gels loaded with whey (Table** 1A), MFGM (Table 1B) and basic proteins (Table 1C), each listed in separate spreadsheets. The database searches that returned a significant match are indicated by MOWSE scores in red font. The fold change was determined by dividing the average spot volume from the mastitis samples with the average volume of the matched spots from the uninfected samples. Changes less than two-fold were deemed to be not significant and assigned “ns”. Click here for file

Additional file 3 **List of 195 proteins detected by either 2D gels or geLC.** The results of Gene Ontology analysis and manual literature curation are shown. Those proteins designated as having a function in host defence are indicated, along with their specific function , with links to relevant literature provided. The GO terms used for a host defence assignment are listed at the bottom of the file. Also listed separately are the human keratins that were detected, presumably as a result of contamination. Click here for file
