# Supplementary material for: Host defence related responses in bovine milk during an experimentally induced Streptococcus uberis infection
Source: Proteome Sci. 2014 Apr 11;12:19. doi: 10.1186/1477-5956-12-19 (PMC4021463; doi:10.1186/1477-5956-12-19)
Supplement: Additional file 1 — Representative 2D gel images of uninfected and mastitic whey, MFGM and basic proteins. The spots indicated with numbers depict those spots subjected to MALDI-TOF analysis. The spot numbers correspond to those listed in Additional file 2. [file 1477-5956-12-19-S1.pptx]

## Slide 1
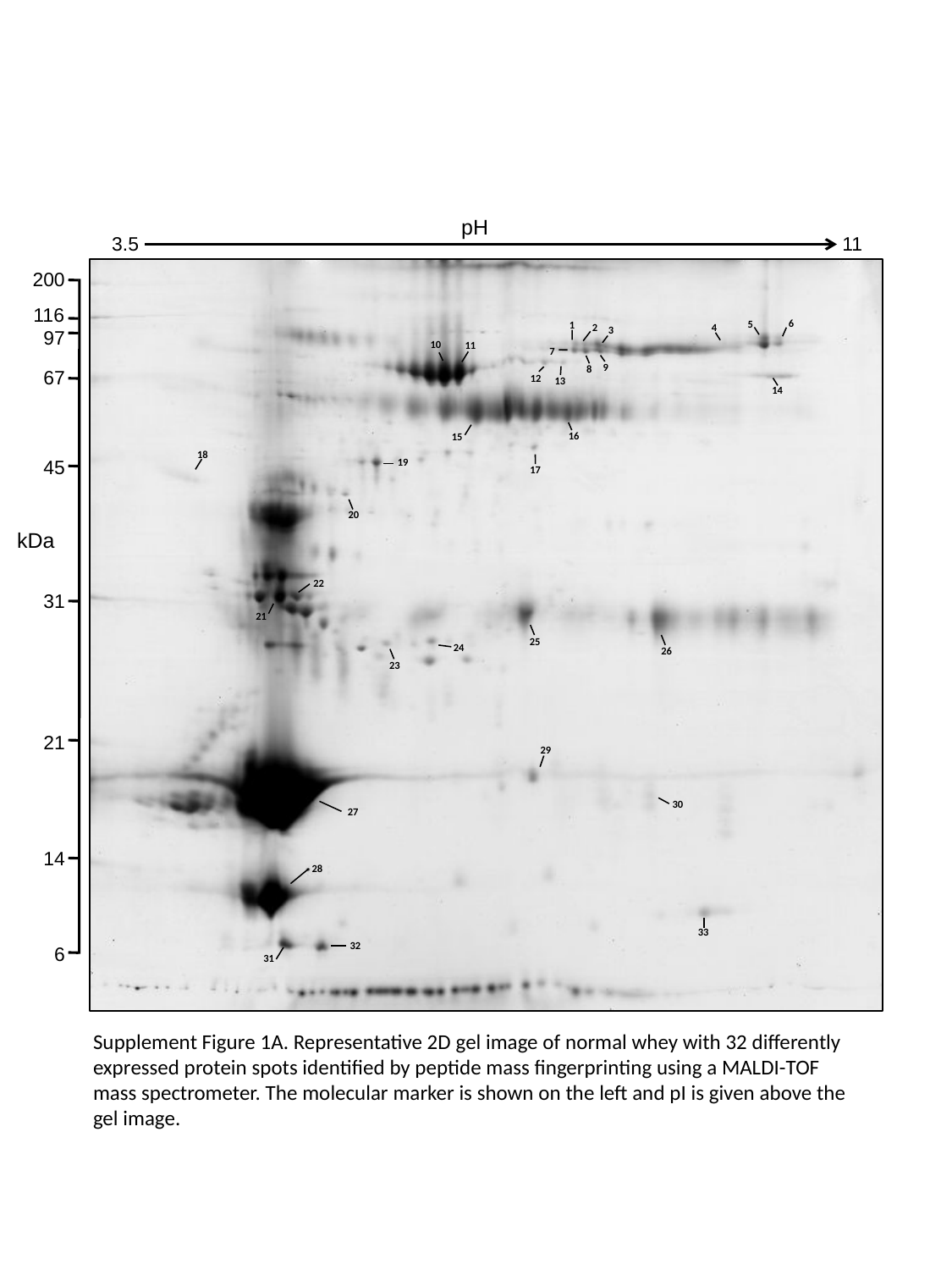

pH
3.5
11
200
116
97
67
45
31
21
14
6
kDa
6
5
1
2
4
3
10
11
7
9
8
12
13
14
16
15
18
19
17
20
22
21
25
24
26
23
29
30
27
28
33
32
31
Supplement Figure 1A. Representative 2D gel image of normal whey with 32 differently expressed protein spots identified by peptide mass fingerprinting using a MALDI-TOF mass spectrometer. The molecular marker is shown on the left and pI is given above the gel image.

## Slide 2
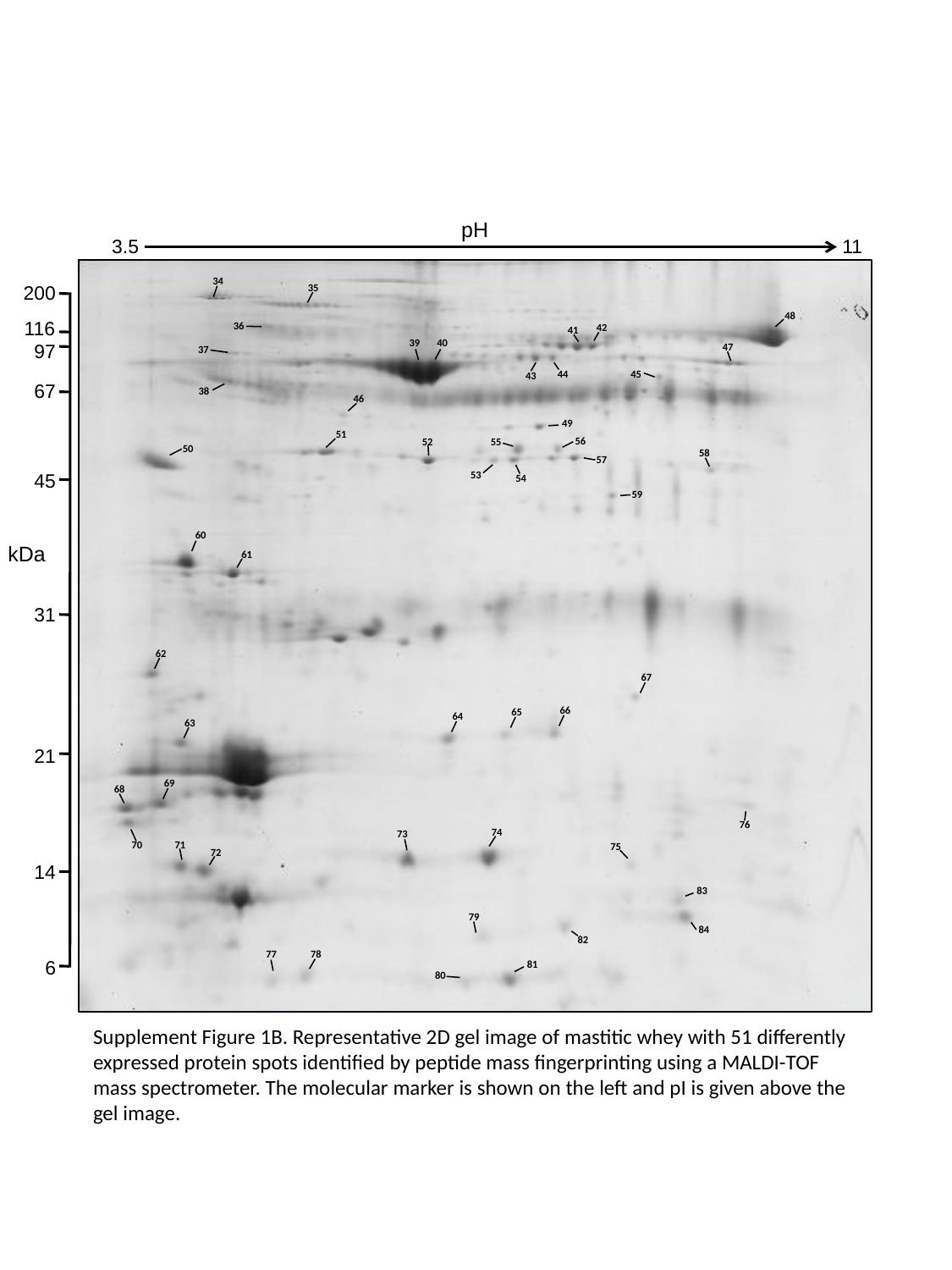

pH
3.5
11
34
200
116
97
67
45
31
21
14
6
kDa
35
48
36
42
41
39
40
47
37
44
45
43
38
46
49
51
56
52
55
50
58
57
53
54
59
60
61
62
67
66
65
64
63
69
68
76
74
73
71
70
75
72
83
79
84
82
78
77
81
80
Supplement Figure 1B. Representative 2D gel image of mastitic whey with 51 differently expressed protein spots identified by peptide mass fingerprinting using a MALDI-TOF mass spectrometer. The molecular marker is shown on the left and pI is given above the gel image.

## Slide 3
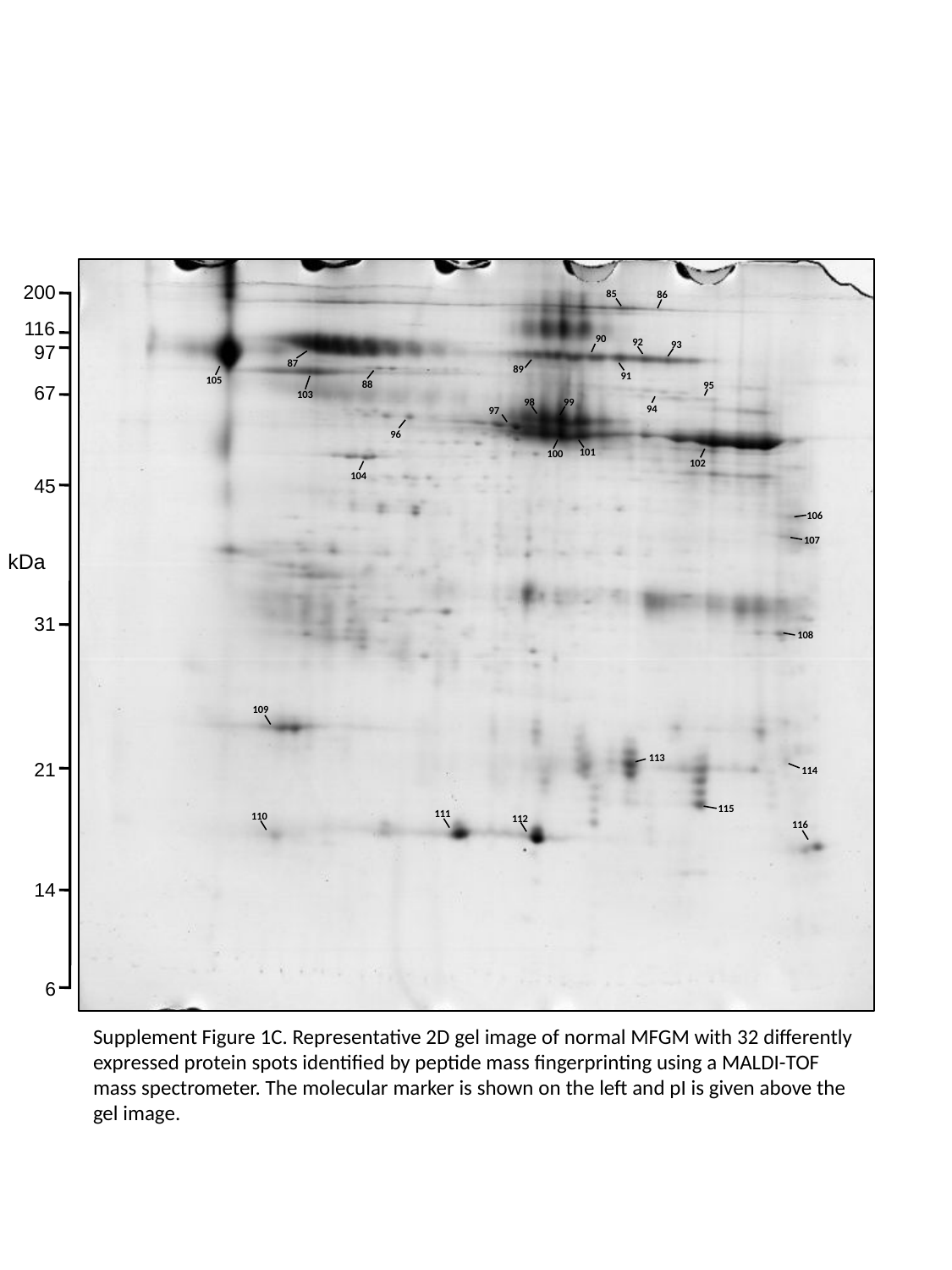

200
116
97
67
45
31
21
14
6
kDa
85
86
90
92
93
87
89
91
105
88
95
103
98
99
94
97
96
101
100
102
104
106
107
108
109
113
114
115
111
110
112
116
Supplement Figure 1C. Representative 2D gel image of normal MFGM with 32 differently expressed protein spots identified by peptide mass fingerprinting using a MALDI-TOF mass spectrometer. The molecular marker is shown on the left and pI is given above the gel image.

## Slide 4
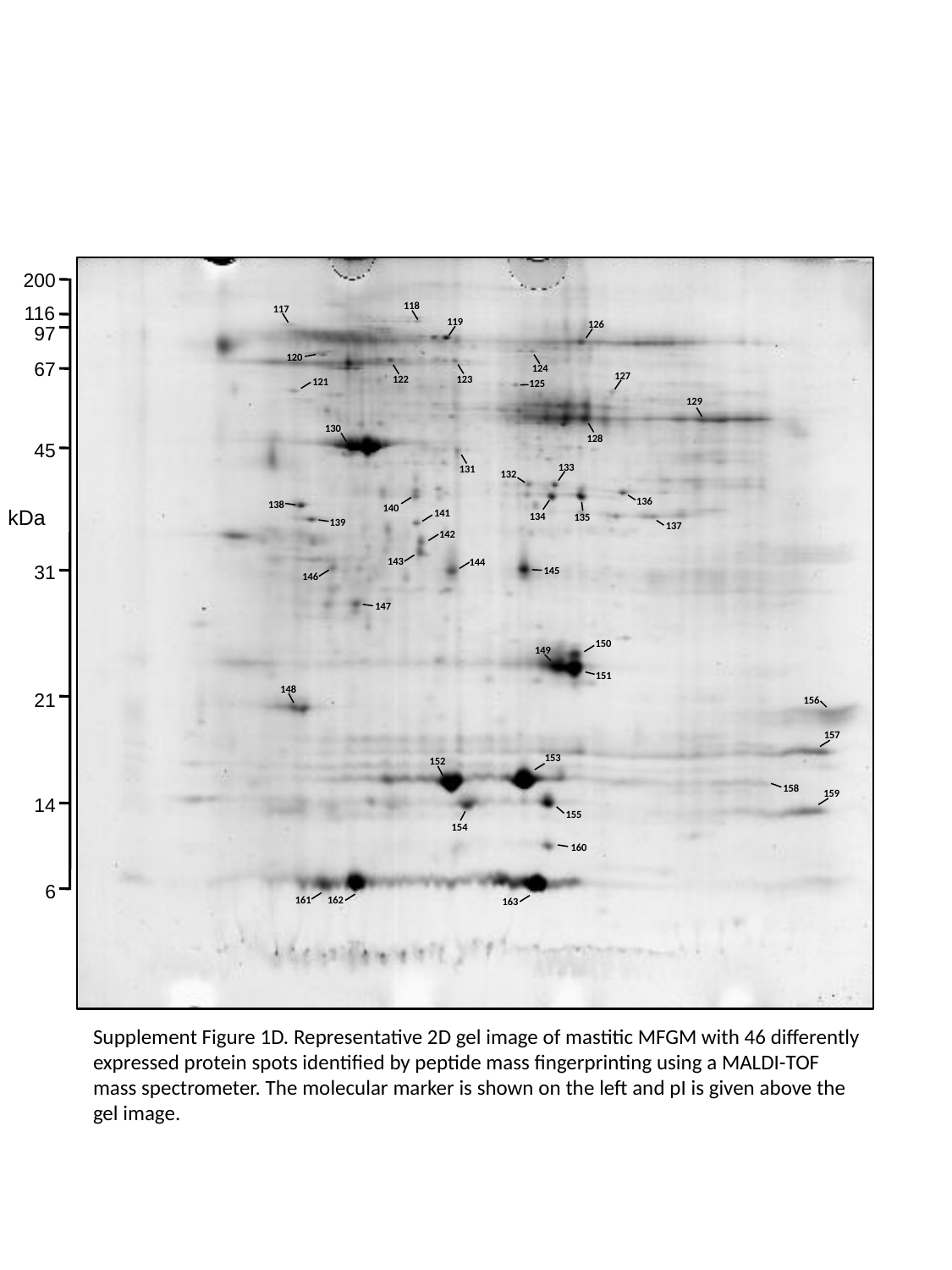

200
116
97
67
45
31
21
14
6
kDa
118
117
119
126
120
124
127
123
122
121
125
129
130
128
133
131
132
136
138
140
141
134
135
139
137
142
143
144
145
146
147
150
149
151
148
156
157
153
152
158
159
155
154
160
161
162
163
Supplement Figure 1D. Representative 2D gel image of mastitic MFGM with 46 differently expressed protein spots identified by peptide mass fingerprinting using a MALDI-TOF mass spectrometer. The molecular marker is shown on the left and pI is given above the gel image.

## Slide 5
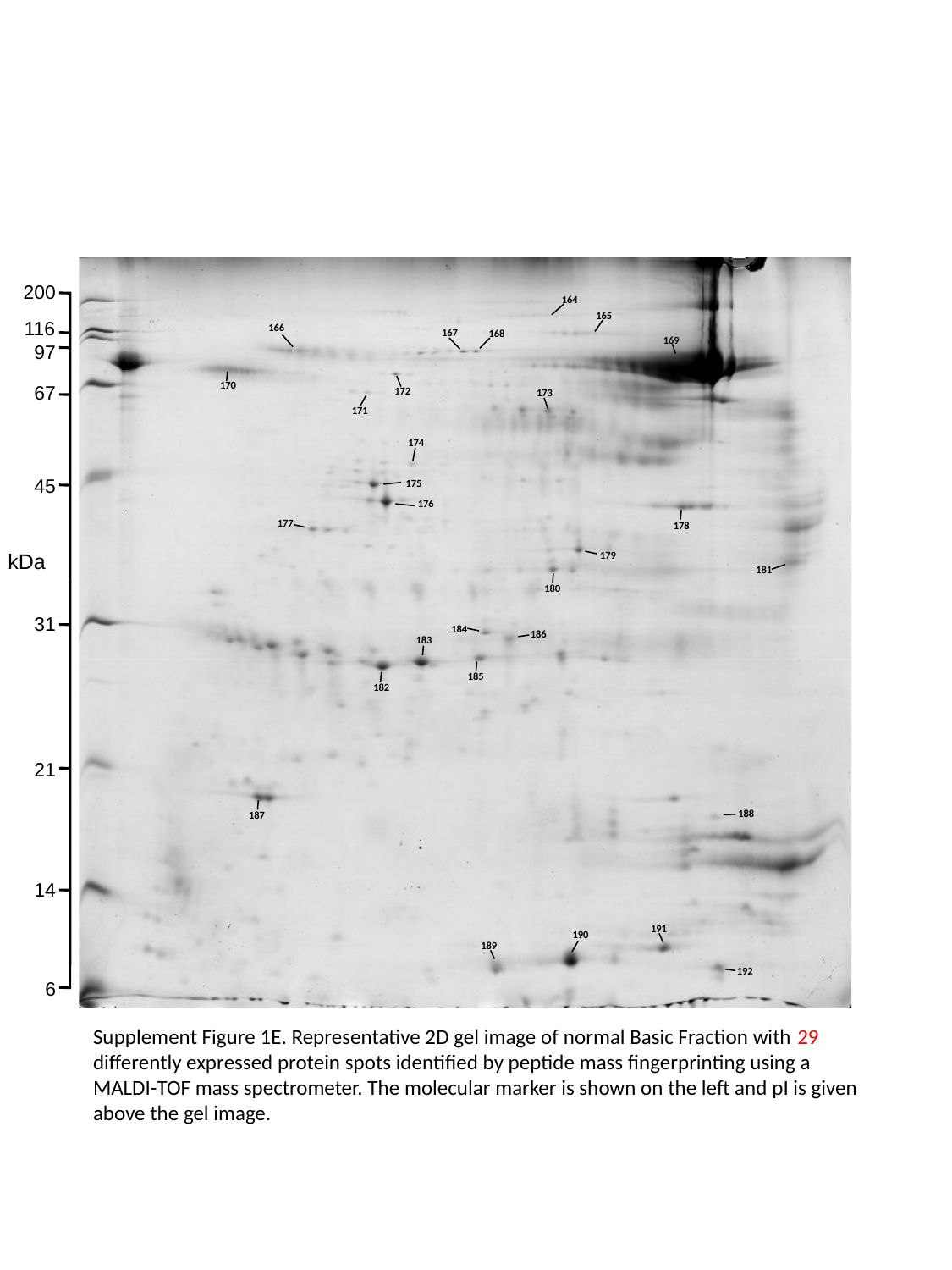

200
116
97
67
45
31
21
14
6
kDa
164
165
166
167
168
169
170
172
173
171
174
175
176
177
178
179
181
180
184
186
183
185
182
188
187
191
190
189
192
Supplement Figure 1E. Representative 2D gel image of normal Basic Fraction with 29 differently expressed protein spots identified by peptide mass fingerprinting using a MALDI-TOF mass spectrometer. The molecular marker is shown on the left and pI is given above the gel image.

## Slide 6
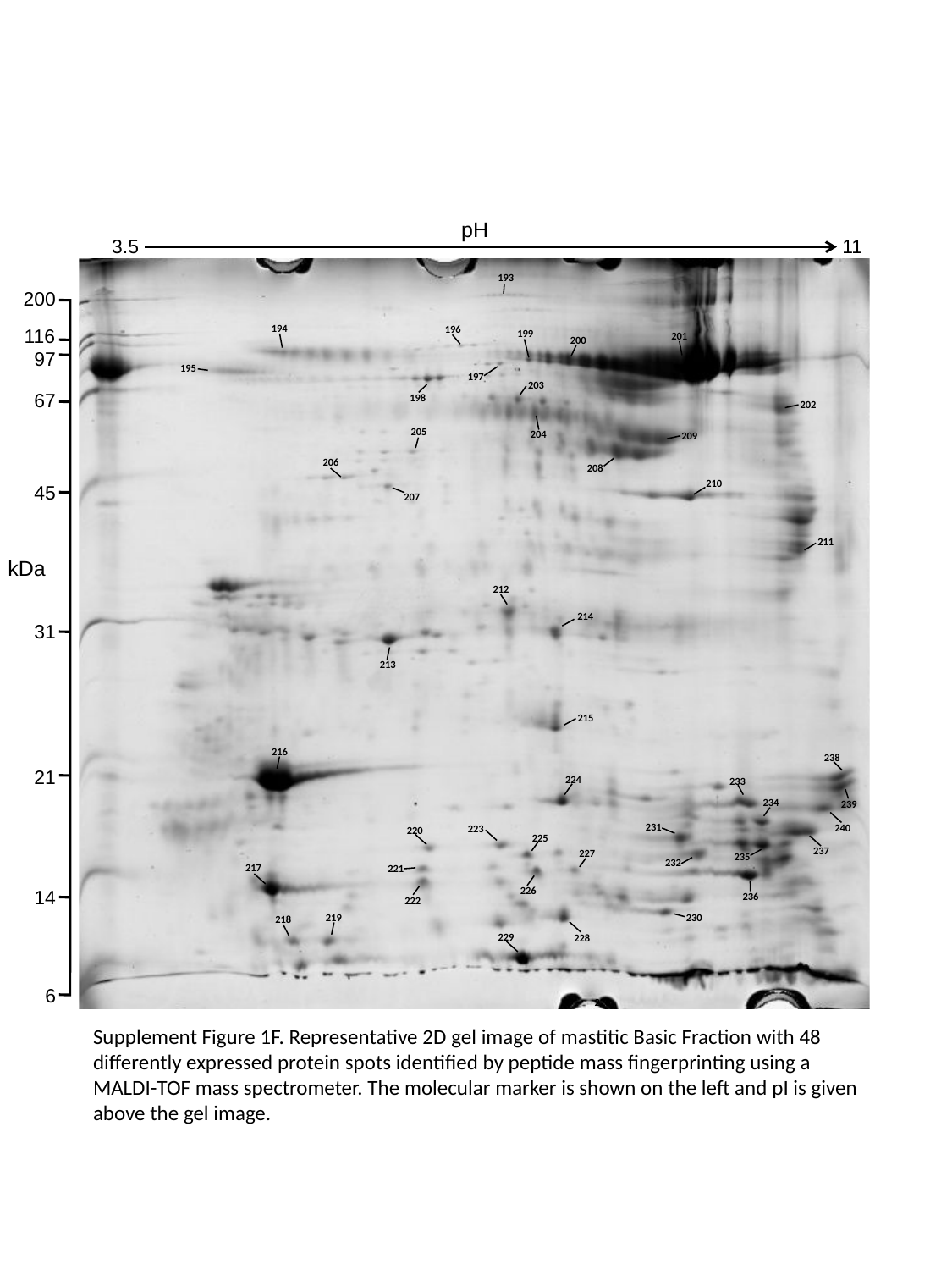

pH
3.5
11
193
200
116
97
67
45
31
21
14
6
kDa
194
196
199
201
200
195
197
203
198
202
205
204
209
206
208
210
207
211
212
214
213
215
216
238
224
233
234
239
231
240
223
220
225
237
227
235
232
217
221
226
236
222
219
230
218
229
228
226
Supplement Figure 1F. Representative 2D gel image of mastitic Basic Fraction with 48 differently expressed protein spots identified by peptide mass fingerprinting using a MALDI-TOF mass spectrometer. The molecular marker is shown on the left and pI is given above the gel image.
